# The biogeographic history of neosuchian crocodiles and the impact of saltwater tolerance variability

**DOI:** 10.1098/rsos.230725

**Published:** 2023-10-04

**Authors:** Sebastian S. Groh, Paul Upchurch, Julia J. Day, Paul M. Barrett

**Affiliations:** ^1^ Department of Earth Sciences, University College London, Gower Street, London WC1E 6BT, UK; ^2^ Department of Genetics, Evolution and Environment, University College London, Gower Street, London WC1E 6BT, UK; ^3^ Quality Enhancement Directorate, Cardiff Metropolitan University, Llandaff Campus, Cardiff CF5 2YB, UK; ^4^ Fossil Reptiles, Amphibians and Birds Section, Natural History Museum, Cromwell Road, London SW7 5BD, UK

**Keywords:** Neosuchia, biogeography, transoceanic dispersal, BioGeoBEARS, Eusuchia

## Abstract

Extant neosuchian crocodiles are represented by only 24 taxa that are confined to the tropics and subtropics. However, at other intervals during their 200 Myr evolutionary history the clade reached considerably higher levels of species-richness, matched by more widespread distributions. Neosuchians have occupied numerous habitats and niches, ranging from dwarf riverine forms to large marine predators. Despite numerous previous studies, several unsolved questions remain with respect to their biogeographic history, including the geographical origins of major groups, e.g. Eusuchia and Neosuchia itself. We carried out the most comprehensive biogeographic analysis of Neosuchia to date, based on a multivariate K-means clustering approach followed by the application of two ancestral area estimation methods (BioGeoBEARS and Bayesian ancestral location estimation) applied to two recently published phylogenies. Our results place the origin of Neosuchia in northwestern Pangaea, with subsequent radiations into Gondwana. Eusuchia probably emerged in the European archipelago during the Late Jurassic/Early Cretaceous, followed by dispersals to the North American and Asian landmasses. We show that putative transoceanic dispersal events are statistically significantly less likely to happen in alligatoroids. This finding is consistent with the saltwater intolerant physiology of extant alligatoroids, bolstering inferences of such intolerance in their ancestral lineages.

## 1. Introduction

Extant crocodiles occur on every continent except Europe and Antarctica, although they are restricted to humid tropical regions [1]. By contrast, extinct members of the Crocodylomorpha were much more widely distributed, with species known from every landmass, even at high latitudes [2]. The 230 Myr history of crocodylomorphs therefore raises intriguing questions regarding how their spatial distributions have changed through time, and the biotic and abiotic factors responsible for these changes. Neosuchia, a major clade within Crocodylomorpha, encompasses all extant taxa (alligators, caimans, crocodiles, gharials), as well as over 300 extinct species [2–4]. The biogeographic history of Neosuchia has received substantial attention, but most previous studies have either read the fossil record literally, to create biogeographic narratives (*sensu* Ball [5]), or have relied on mapping fossil occurrences on to phylogenetic trees (e.g. [6,7]). Although these qualitative approaches have yielded some potentially important insights into neosuchian biogeographic history, and have generated numerous hypotheses, these methods are now regarded as outdated (e.g. [8]) owing to the development of more sophisticated analytical methods [9–14]. Some of these quantitative phylogenetic biogeographic methods have been applied to neosuchian clades, including: the use of ‘tree reconciliation analysis' to identify vicariance in Cretaceous crocodyliforms [15]; statistical dispersal vicariance analyses (S-DIVA) that investigated the biogeographic patterns of South American Miocene crocodylians [16] and Dyrosauridae [17]; and BioGeoBEARS (BGB) studies of Caimaninae [18] and *Crocodylus* [19]. As a result of these studies, it is generally agreed that vicariance played an important role during neosuchian evolution [7,15,20,21]. Despite this progress, many aspects of neosuchian biogeography remain obscure or disputed. For example, the area where neosuchians first evolved is still debated, because the Jurassic record of non-pelagic taxa is poor and the major groups within Neosuchia all have different putative geographical origins [22]. Eusuchia potentially originated either in Europe [23] or Gondwana [24], and Early Cretaceous specimens from Asia [25,26] suggest that the latter area might also be a possible centre of origin. Tethysuchia evolved either in Africa [17,27–29] or North America [30] and Goniopholididae either in North America or central Asia [30,31]. These issues are further complicated by the differing degrees of saltwater tolerance within Neosuchia. Saltwater tolerance varies strongly among both extant and extinct species, enabling marine dispersal routes for some taxa but not others. Several groups were apparently saltwater tolerant, or even marine, such as Dyrosauridae [29], Tomistominae [20,32] and Crocodylinae [1,2,33,34], and such tolerance also seems probable for the extinct members of some living saltwater intolerant groups such as the gavialoid clade Gryposuchinae [2,35]. By contrast, extant members of Alligatorinae and Caimaninae have very low saltwater tolerance and the physiological status of their ancestors is unclear [6,16,36]. Thus, many aspects of neosuchian biogeography require further investigation, ranging from the detailed histories of individual lineages through to the relative contributions of factors such as vicariance and transoceanic dispersal. Moreover, there have been few previous attempts to assess the sensitivity of inferred neosuchian biogeographic patterns to variations in the input data (e.g. different phylogenetic topologies) and the methods selected for analysis.

The current study addresses several of the gaps in our knowledge of neosuchian biogeography as outlined above. We have built datasets consisting of two recent, extensive phylogenies for Neosuchia [37–39] and information on the geographical distributions of their terminal taxa. We then apply two recently developed techniques for estimating the ancestral geographical ranges of taxa using time-scaled phylogenies: BGB [9,10]; and Bayesian ancestral location estimation (BALE) [12]. Uniquely, compared to all previous studies, we attempt to estimate the relative frequency of transoceanic dispersal among the ancestors of several key clades, in order to assess the potential impact of saltwater tolerance/intolerance on biogeographic history. Finally, we place our results in the wider context of neosuchian biogeography, evaluating previously proposed hypotheses and presenting new ones.

## 2. Material and methods

### 2.1. Neosuchian spatio-temporal occurrence data

Our biogeographic analyses require data on the spatio-temporal distributions of the terminal taxa in our phylogenies. For this purpose, we downloaded occurrence data (including palaeocoordinates) for Neosuchia (and also Aegyptosuchidae, which are potentially part of Neosuchia [40]) from the Paleobiology Database (PBDB) (https://paleobiodb.org; accessed 8 July 2021) for fossil taxa, and the Global Biodiversity Information Facility (GBIF) for extant taxa [41]. We further excluded taxa of uncertain taxonomic identification (such as *Crocodylus* sp.) and with very little material (e.g. identification based on isolated teeth). For the *k*-means clustering analysis (see below) we excluded all taxa known to be primarily marine (for example, *Terminonaris*), because the focus of this study is on ancestral area estimation relating to organisms occupying landmasses. This dataset comprises 1132 occurrences of 323 species and can be found in the Dryad Digital Repository [42, S1]. The datasets for use with the two phylogenetic trees, including all taxa included in the trees, can be found in [42, S2].

### 2.2. Phylogenetic trees and time scaling

We used two different sets of phylogenies for the biogeographic analyses: (i) the supermatrix-based neosuchian phylogenies from Groh *et al*. [37] as modified by Groh *et al*. [38] (hereafter termed the ‘G22’ trees); and (ii) the neosuchian portion of the supertree of Stockdale & Benton [39] (hereafter termed the ‘SB21’ tree). For G22, we selected the phylogeny resulting from the extended implied weighting analysis (*k* = 3) of the character set including continuous characters [37]. For the Stockdale & Benton [39] analysis, we chose the tree excluding Thalattosuchia for comparability between the two phylogenies. Both tree sets were time-scaled using cal3 [43] and the fossilized birth–death (FBD) model [44,–46], as described in detail in Groh *et al.* [38]. For both phylogenies, a time-scaled tree topology was created using the mean node age estimations from each method. Because neither of the biogeographic methods work with zero-branch lengths, we used a minimum branch length method and any zero-length branches in the trees were rescaled to have a length of 0.1 Myr. The four time-scaled trees are presented in [42, S2].

### 2.3. Bayesian ancestral location estimation

The BALE approach was introduced by O'Donovan *et al.* [12], although the name for this method was coined by [8]. Unlike BGB, which relies on pre-defined geographical areas (see below), BALE employs a species' palaeocoordinates (i.e. palaeolatitude and palaeolongitude) as a basis for estimating ancestral areas in a three-dimensional space, using a Bayesian framework. Essentially, the method works by taking the geographical coordinates of each of the terminal taxa in a phylogeny and treating these as ‘trait data’. Bayesian approaches are then applied in order to estimate the value of these traits at each node [12]. The coordinates of the taxa in our phylogenies were derived from the PBDB dataset described above, including additional screening ensuring that only valid identifications were counted as location. However, it was not feasible to treat every occurrence point as a tip in the phylogenetic tree, as done by O'Donovan *et al.* [12], because our tree topologies contain many extant taxa and a large proportion of non-singleton extinct taxa, which thus have multiple occurrences. Therefore, we determined the geographical midpoint for each multi-occurrence taxon that was part of our phylogeny using geomidpoint method A (http://geomidpoint.com/calculation.html; accessed 6 August 2021). In this method, the latitudes and longitudes (or palaeolatitudes and palaeolongitudes in the case of extinct taxa) are converted into Cartesian coordinates. Their midpoint is then calculated taking into account the curved surface of the Earth. The complete list of coordinates used in the analyses is available in [42, S1]. The BALE method was implemented in BayesTraits v. 3.0.1 [47]. This was applied to each tree five times, with 10 000 000 iterations per run, and a burn-in of 1 500 000. R 4.2.0 [48] was used to concatenate the results and calculate the median palaeolatitude and palaeolongitude (with confidence intervals) for each node. Coordinates were matched onto a model of the Earth at the respective age of each node using the PALEOMAP PaleoAtlas [49] in GPlates 2.0.0 [50,51] in order to determine the area of origin for each neosuchian group of interest.

### 2.4. BioGeoBEARS

#### 2.4.1. The basic methodological approach

BGB, developed by Matzke [9,52] is a package that provides a single framework in which different biogeographic models for ancestral area estimation can be applied and compared. There are three main models: dispersal, extinction, cladogenesis (DEC [53,54]); a maximum-likelihood implementation of dispersal-vicariance analysis (DIVALIKE, based on Ronquist [55]); and BAYAREALIKE (based on Landis *et al.* [56]). BGB methods use maximum likelihood to estimate the area(s) occupied at each ancestral node of a dated tree, incorporating prior probabilities of potential changes in the ranges of ancestral areas at each node based on different rules imposed by each of the three main models. All three models allow dispersal and extinction to occur along tree branches. DEC permits both narrow and subset sympatry, and a narrow form of vicariance (in which one of the two daughter lineages occupies only one of the available areas), at each node [54]. DIVALIKE permits only a narrow form of sympatric speciation, but both daughter species can inherit more than one area from their immediate common ancestor when vicariance is estimated [55]. BAYAREALIKE prohibits vicariance: instead, daughter lineages simply inherit the full set of areas occupied by their immediate common ancestor [9,56]. Matzke [9,10] added the option to include founder event speciation (parameter J) to each of the three main models. Thus, the BGB package estimates biogeographic history by applying six different models, with log-likelihood ratio tests and the Akaike information criterion (AIC) used to determine which model(s) best fit the data.

#### 2.4.2. Identifying suitable geographical units

**Figure 1.**
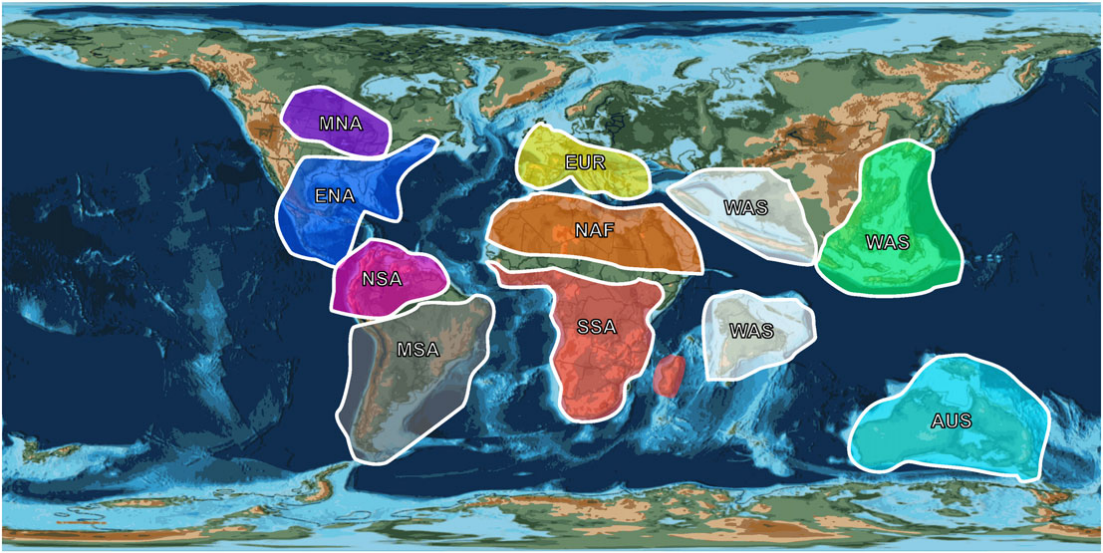
A map of the 10 geographical areas used in the BioGeoBEARS analysis. The map is based on a reconstruction of the Earth 66 Ma, created in GPlates 2.0.0 [50,51] using the PALEOMAP PaleoAtlas [49]. The 10 areas were drawn according to the clusters detected with the *k*-means analysis.

Most analytical biogeographic methods, including BGB, require that taxa be assigned to one or more geographical units, sometimes referred to as ‘areas of endemism’ [9–11,15,57,58]. Traditionally, studies of large-scale and long-term historical biogeographic patterns among terrestrial vertebrates have based such units on modern continental areas such as ‘North America’, or have developed units (e.g. Laramidia) based on palaeogeographic reconstructions [4,15,58–63]. However, this approach does not always reflect the true boundaries between distinct or endemic biotas, especially in ‘deep time’ when continental configurations were different and other geographical features existed, such as ephemeral continental seaways or former mountain ranges [11]. In particular, climatic zones and the complex relationship between biome distributions and the ecological requirements of individual clades, could have created dispersal routes or barriers that are not readily apparent from an inspection of palaeogeographic maps. An alternative approach is to use the distributions of the taxa themselves to identify clusters that potentially correspond to distinct biotas. To this end, we employed the multivariate *k*-means clustering approach, proposed by Button *et al.* [11], to establish geographical distribution areas for neosuchians throughout their evolutionary history. The palaeocoordinate data for neosuchian occurrences (see §2.1) was first binned at epoch level in order to reduce the distributional ‘noise’ generated by long-term plate tectonic motions [11]. The occurrences during the Jurassic were too sparse for statistically meaningful analysis, so taxa were allotted to one of four time bins: Early Cretaceous, Late Cretaceous, Palaeogene and Neogene + Quaternary. Following the analytical protocol described in Button *et al.* [11], we applied *k*-means clustering, with *k* (corresponding to the number of clusters being explored) varying between 5 and 15 for each epoch. These analyses were carried out in R 4.2.0 [48], with 10 000 replicates for each value of *k,* and 10 random starts. The performance of a given *k* value is measured as follows:

$$\mathrm{PVE}=\frac{\mathrm{BCSS}}{\mathrm{TSS}},$$

where PVE is the proportion of the total variance explained by the resolved clusters; BCSS is the between clusters sum of squares; and TSS is the total sum of squares (see Button *et al*. [11],[42, note 3]). Button *et al*. [11] used a cut-off PVE value of 98%: that is, cases where the proposed *k* value resulted in less than 98% of the variance being explained, were rejected. The results of our *k*-means clustering analyses are shown in table 1. Even with a 98% cut-off value, there is more than one viable *k* value for each time bin, so these analyses have not suggested a unique set of ‘areas of endemism’ for use in BGB analyses. Button *et al*. [11] faced a similar issue and selected a final set of 10 areas based on their consistency across time bins and their conformation to pre-existing biogeographic provinces or palaeogeographic units. In order to apply these criteria to our results, the locations of the geographical clusters were plotted on to palaeomaps corresponding to the midpoint of each epoch, using the PALEOMAP PaleoAtlas [49] in GPlates 2.0.0 [50,51], and a bespoke script written by the authors, using functions from the R packages rgdal [64] and ggplot2 [65]. This script is available in [42, information file S1]. This resulted in the identification of 10 geographical areas for use in our BGB analyses, as follows (figure 1): AUS, Australia; EAS, east Asia (including southeast Asia and Mongolia); ENA, eastern North America; EUR, Europe; MNA, middle North America; MSA, middle and southern South America; NAF, North Africa; NSA, Central America and northern South America; SSA, central and southern Africa; WAS, west and central Asia (including India, because the Indian species in our phylogenies only occur there after India collided with the Asian continent).

**Table 1.** Summary of the results of the multivariate *k*-means analyses; this shows the total variance (in %) explained by the different numbers of clusters (i.e. the PVE value) for each epoch. (All values greater than 98% are shaded. See main text for details.)

number of clusters (*k*)											
	5	6	7	8	9	10	11	12	13	14	15
Early Cretaceous	96.7	97.5	97.6	98.5	98.7	99.0	98.9	99.2	99.3	99.5	99.7
Late Cretaceous	94.9	96.7	96.9	98.0	98.2	97.7	98.9	98.9	98.8	98.9	99.2
Palaeogene	96.5	97.7	98.1	98.4	98.6	98.8	99.0	99.0	99.2	99.4	99.5
Neogene + Quaternary	95.0	95.5	97.5	97.7	98.0	98.5	98.2	98.8	98.7	98.9	99.1

#### 2.4.3. Constraints on dispersal probability

Although ancestral areas can be estimated with the methods contained in BGB using just a dated phylogeny and information on the geographical units occupied by terminal taxa, the package also offers several ways to incorporate palaeogeographic information, such as the connectedness of areas or their relative distances from each other [9,10]. Depending on what is known, or hypothesized, about the dispersal abilities of the target organisms, such constraints can be used to provide more realistic models of the biogeographic evolution of any given clade. Such constraints are held in matrices that specify how the probability of dispersal between each pair of areas should be up- or down-weighted (Matzke [9]; see also Poropat *et al*. [62]). Given that palaeogeography has changed through time, BGB allows use of a series of different matrices, each capturing the particular palaeogeographic relationships pertaining to a given time window.

As noted above, many crocodylomorph lineages (including several neosuchian clades) display evidence of saltwater tolerance and therefore had the potential for transoceanic dispersal. Consequently, it would be inappropriate to constrain neosuchians to dispersal routes limited to intervening land connections, as has been justified for several dinosaur groups [62,63,66,67]. Nevertheless, it also seems unrealistic to suggest that dispersal probabilities are unaffected by geographical distance. We therefore employed two different approaches within BGB: (i) an unconstrained analysis that did not place any limits on dispersal probability between our 10 geographical areas; and (ii) a distance-based approach that modified the dispersal probabilities between these areas, depending on their physical proximity during different time periods [68]. The unconstrained results are useful as a baseline, indicating the biogeographic history that is estimated with the minimum number of assumptions. The constraint analysis is potentially more ‘realistic’, and comparison between it and the unconstrained analysis can indicate the impact of our additional assumptions.

For the distance-based approach, we designated seven time bins: 0–30 Ma, 30–60 Ma, 60–90 Ma, 90–120 Ma, 120–150 Ma, 150–180 Ma and 180–230 Ma. For each time bin, the midpoint was taken (e.g. 205 Ma for the 180–230 Ma bin), and the position of landmasses at each temporal midpoint was then visualized using PALEOMAP PaleoAtlas [49] in GPlates 2.0.0 [50,51]. We then calculated the midpoint palaeocoordinates of each of our geographical areas (see §2.4.2, above) and calculated the geographical distances (in kilometres) between them for each timebin. All distances were scaled by dividing them by the shortest recorded distance (1682 km), and these relative distance values were then used to construct the distance matrices. Consequently, in the constrained analyses, the probability of dispersing from one geographical area to another is inversely proportional to the distance between the midpoints of those areas, and these probabilities are not affected by whether dispersal is entirely via land or requires crossing an ocean.

The tree files, distance matrices, time periods file (which designate the seven time bins) and geographical range files are presented in [42, S2]. All analyses were performed in R 4.2.0 [48].

### 2.5. Transoceanic dispersal and saltwater tolerance

If extinct members of Alligatoroidea were saltwater intolerant, like their extant representatives, then it is predicted that biogeographic analyses should estimate fewer instances of transoceanic dispersal in this clade when compared to those neosuchian clades with saltwater tolerance. The results of BGB analyses can be used to identify when and where dispersals have occurred and cross-referencing of these events with palaeogeographic reconstructions can then reveal whether dispersal occurred via land or across an ocean. It is therefore feasible to estimate the number of putative transoceanic dispersal events within different neosuchian clades, and to evaluate statistically whether there are skews that conform to these predictions. Skews in the frequency of transoceanic dispersal events, in saltwater tolerant and intolerant clades, can be tested using Pearson's *χ*^2^-tests. Such tests determine whether the expected number of transoceanic dispersals (if there is no skew between taxon groups) is statistically distinguishable from the observed values. Calculation of the expected number of transoceanic dispersals is complicated by the fact that different groups have different numbers of taxa. An analogous issue of uneven sampling has been encountered by studies that have used *χ*^2^-tests to examine environmental associations among dinosaur groups (e.g. [69–71]). We therefore follow these studies in using a modified version of the approach proposed by Waite [72]. The expected number of transoceanic dispersals is given by

$$E_{\mathrm{toct}}=\frac{N_{\mathrm{to}}\times N_{\mathrm{ct}}}{N_{T}}$$

and

$$E_{\mathrm{toci}}=\frac{\left(N_{\mathrm{to}}\times N_{\mathrm{ci}}\right)}{N_{T}},$$

where *E*_toct_ is the expected number of transoceanic dispersals in a saltwater tolerant group, and *E*_toci_ is the expected number in an intolerant group; *N*_to_ is the total number of putative transoceanic dispersal events across both groups; *N*_ct_ and *N*_ci_ are the numbers of nodes in the saltwater tolerant and intolerant groups, respectively; and *N_T_* is the total number of nodes across the groups being compared. As a hypothetical example, suppose group A is three times the size of group B, such that A has 30 nodes and B has 10. Suppose also that, across the two clades, there are 20 transoceanic dispersal events. A non-skewed distribution would be one in which there are three times as many transoceanic dispersals in A as there are in B. This distribution is given when the equations above are applied: for group A, the expected value would be given by (20 × 30)/40 = 15, and for group B this would be (20 × 10)/40 = 5. A *p*-value of 0.05 is set as the cut-off for statistical significance.

In several cases, it is unclear whether dispersal happened in a transoceanic manner or via the use of a longer hypothetical terrestrial dispersal route. For each distinct biogeographic estimation (i.e. distance-based G22 with cal3 and FBD, unconstrained G22 with FBD and distance-based SB21 with cal3; note, the other biogeographic analyses delivered identical results) we therefore analysed two scenarios which assumed the minimum and maximum numbers of potential transoceanic dispersals. We compared the number of putative transoceanic dispersals within Alligatoroidea (the saltwater intolerant group) to that within the remaining Crocodylia (a mostly saltwater tolerant group). For each set of maximum or minimum number of putative transoceanic dispersals, we calculated the *χ*^2^-value to test whether there was a significant difference between the saltwater tolerant and intolerant groups. Because we tested different scenarios for each calculation (i.e. the tests were not applied to the same, or strongly overlapping, datasets multiple times), there was no need to adjust *p*-values using the false discovery rate [73].

## 3. Results

### 3.1. Ancestral area estimation

For all BGB analyses (both G22 and SB21, unconstrained and distance-based), the DEC + J model had the best fit to the data, with AIC and AICc weights ranging from 0.95 to 1.0 across analyses (see [42, S2] for details of the statistical values resulting from all analyses). The *p*-values (less than 0.0001 for each analysis) indicate that DEC + J is a significantly better fit to the data than DEC. There has been criticism of the validity of DEC + J results [74], but this has been contested [75]. There is considerable agreement across the G22 and SB21 trees, for both the unconstrained and constrained analyses, in terms of the ancestral area estimations, although there is less congruence among clades that have diverged more recently (see below).

The results of the BALE analyses showed some agreement between topologies scaled with the same time-scaling method. The estimated ancestral areas are often situated on similar larger landmasses, such as continents, but may differ in terms of which of our 10 geographical areas are estimated. In several cases (one to four major groups per analysis, in particular those time-scaled using FBD), BALE placed the origin of a group in the middle of a large water body rather than a land area.

The ancestral areas estimated by BGB and BALE are summarized for the major clades in table 2. Despite some key differences (noted below) these two biogeographic approaches generally produced relatively congruent results. The BALE analyses suggest various origins of Neosuchia in North America (mostly eastern North America). By contrast, the BGB analyses unanimously identify Europe as the ancestral area for this clade.

**Table 2.** A list of ancestral areas for major neosuchian clades, as estimated by the different biogeographic analyses. (Area abbreviations are as follows: AUS, Australia; EAS, east Asia (including southeast Asia and Mongolia); ENA, eastern North America; EUR, Europe; MNA, middle North America; MSA, middle and southern South America; NAF, North Africa; NSA, Central America and northern South America; SSA, central and southern Africa; WAS, west and middle Asia (including India, because the species in our trees only occur there after India joined the Asian continent).)

	G22					SB21		
	BALE		BGB			BALE		BGB
	cal3	FBD	distance		unconstrained	cal3	FBD	all
			cal3	FBD				
**Neosuchia**	between India and Antarctica	ENA	EUR	EUR	EUR	ENA/MNA	ENA	EUR
**Tethysuchia**	—	—	—	—	—	west Asia	EUR	ENA
**Dyrosauridae**	NAF	NAF	ENA	ENA	ENA	NAF	NAF	NAF
**Goniopholididae**	MNA	MNA	EUR	EUR	EUR	ENA	ENA	EUR
**Eusuchia**	EUR	ENA/NAF/EUR	EUR	ENA	EUR	btw North Africa and Europe	Arabian peninsula	EUR
**Hylaeochampsidae**	EUR	EUR	EUR	EUR	EUR	—	—	—
**Alligatoroidea**	MNA	north North America	MNA	MNA	MNA	MNA	North America	MNA
**Diplocynodontinae**	EUR	EUR	EUR	EUR	EUR	EUR	EUR	EUR
**Globidonta**	MNA	North America	MNA	MNA	MNA	MNA	MNA	MNA
**Alligatoridae**	—	—	—	—	—	Central America	ENA	MNA
**Alligatorinae**	—	—	—	—	—	ENA	MNA/ENA	MNA
**Caimaninae**	MNA	MNA	NSA	NSA	NSA	NSA	Central America	NSA
**Crocodyloidea**^a^	MNA	Greenland	MNA	MNA	MNA	northern North America	Greenland	MNA
**Crocodylidae**	—	—	—	—	—	AUS	middle of Indian Ocean	AUS
**Mekosuchinae**	—	—	—	—	—	AUS	AUS	AUS
**Crocodylinae**	EAS	west Asia	NAF	NAF	EAS	NAF	Arabian peninsula	SSA
**Gavialoidea**	ENA	ENA	ENA/EUR	ENA	ENA/EUR	Greenland	EUR	EUR
**Gavialinae**	NAF	EUR	NAF	NAF	NAF	—	—	—
**Tomistominae**	EUR	EUR	EAS	EAS	EAS	—	—	—

^a^Includes Gavialidae in the SB21 tree.

The area of origin of Tethysuchia is variously estimated by BALE as either western-most Asia (SB21, cal3) or eastern Europe (SB21, FBD), whereas all SB21 BGB analyses suggest eastern North America (note, there is no equivalent clade in the G22 tree because Tethysuchia is paraphyletic, see Groh *et al*. [38]). BALE estimates northern Africa as the area of origin for Dyrosauridae: this is in partial agreement with the BGB analyses in which North Africa is suggested by SB21, whereas G22 estimates eastern North America (via Europe) (table 2). The area of origin for Goniopholididae is estimated as North America, either middle North America (G22) or eastern North America (SB21) by BALE: by contrast, Europe is suggested by all BGB analyses.

For Eusuchia, BALE estimates the ancestral area as Europe (G22, cal3), the Arabian Peninsula (SB21, FBD) or somewhere between North Africa, Europe and eastern North America (G22, FBD and SB21, cal3). This is in broad agreement with the BGB results: all but one analysis postulates Europe as the ancestral area for Eusuchia, whether Thalattosuchia are included or not in Neosuchia (table 2). The exception is the distance-based BGB analysis of the G22 FBD tree, which estimates eastern North America as the ancestral area for Eusuchia.

There is no Hylaeochampsidae clade in the SB21 trees, but the G22 BGB and BALE analyses all support Europe as its ancestral area. Crocodylia is equivalent to Eusuchia in the G22 trees. In all SB21 trees, middle North America emerges as the most likely place of origin for Crocodylia. Within Crocodylia, Gavialoidea originated either in northern/northeastern east North America (G22), Greenland (SB21, cal3) or southeastern Europe (SB21, FBD) according to BALE. A similar result is supported by the BGB analyses, with Europe (SB21), eastern North America (G22 FBD, distance-based), or equal probabilities for both these areas (all other G22 analyses), being postulated as the area of origin. Gavialinae and Tomistominae are only present as distinct groups in the G22 trees, so no conclusions can be drawn from the SB 21 trees. BALE estimates Europe as the area of origin for both of these clades in most cases, except for the cal3 G22 tree which supports northeastern North Africa for Gavialinae (table 2). BGB estimates the ancestral area as North Africa for Gavialinae (in line with the G22 cal3 BALE result) and East Asia for Tomistominae, across all analyses.

BALE and BGB agree that Europe is most probably the ancestral area of Diplocynodontinae. BALE and BGB unanimously agree on middle North America as the ancestral area for Alligatoroidea, Alligatorinae and Globidonta, in all G22 and SB21 trees (table 2). For Caimaninae, the BALE analysis of the SB21 cal3 tree identifies northern South America as the area of origin, whereas the SB21 FBD tree suggests Central America, and the G22 trees support western middle North America. The BGB results unanimously support northern South America as the area of origin of this clade, in line with the SB21 cal3 BALE analysis.

Crocodyloidea is estimated to have originated in middle North America by all BALE and BGB analyses. Crocodylidae and Mekosuchinae are not present as distinct clades in the G22 tree. However, the BALE and BGB analyses of SB21 all agree on Australia as the ancestral area for both of these clades. By contrast, our analyses were unable to provide a decisive estimate of the area of origin for Crocodylinae: BALE supports Asia (G22), North Africa (SB21, cal3) or the southeastern Arabian Peninsula (SB21, FBD); all BGB SB21 analyses suggest central and southern Africa, whereas the G22 analyses estimate North Africa (distance-based analysis) and east Asia (unconstrained analysis) (table 2).

Finally, although the above summary indicates strong agreement between BGB and BALE (at least to broad geographical areas), there remain discrepancies that appear to be driven by phylogenetic topology. There are considerable differences between the estimated ancestral areas for the G22 and SB21 trees, with most of these disagreements occurring outside Alligatoroidea. As noted above, there are several clades in one of these trees that are not present (or cannot be unambiguously identified) in the other, as well as early branching taxa present in one tree and not the other (such as *Jiangxisuchus nankangensis* in the SB21 phylogeny). It is therefore not surprising that phylogenetic topology is linked to many of the more equivocal aspects of our results. Why there should be more agreement between the SB21 and G22 trees with regard to alligatoroids is examined further in the Discussion.

### 3.2. Transoceanic dispersals

Assuming the minimum necessary number of transoceanic dispersals according to our estimated ancestral areas, Alligatoroidea has significantly fewer transoceanic dispersals than would be expected by chance when compared to the remaining crocodylian groups (*p* < 0.05). These results hold true across all G22 trees, with the exception of the distance-scaled FBD tree. A similar pattern emerges across the SB21 trees, with Alligatoroidea consistently showing significantly fewer transoceanic dispersal events than expected. For ‘non-alligatoroid crocodylians', the G22 trees do not produce any significant results, but the SB21 trees do provide support for significantly more transoceanic dispersal than expected by chance (table 3).

**Table 3.** Chi-square and associated *p*-values for testing whether there is a significant difference in the expected versus observed frequency of transoceanic dispersals in Alligatoroidea and Crocodyloidea, with the minimum and maximum number of possible dispersal events considered. (The asterisk denotes significant *p*-values.)

		minimum dispersal		maximum dispersal	
		dispersal events within Alligatoroidea	dispersal events within Crocodyloidea	dispersal events within Alligatoroidea	dispersal events within Crocodyloidea
G22 distance	cal3	*χ*^2^ = 4.22, *p* = 0.040*	*χ*^2^ = 1.25, *p* = 0.264	*χ*^2^ = 2.72, *p* = 0.099	*χ*^2^ = 0.81, *p* = 0.368
G22 distance	FBD	*χ*^2^ = 3.42, *p* = 0.064	*χ*^2^ = 1.02, *p* = 0.313	*χ*^2^ = 3.30, *p* = 0.069	*χ*^2^ = 0.98, *p* = 0.322
G22 unconstrained	FBD	*χ*^2^ = 4.59, *p* = 0.032*	*χ*^2^ = 1.36, *p* = 0.244	*χ*^2^ = 3.70, *p* = 0.054	*χ*^2^ = 1.10, *p* = 0.294
SB 21 distance	cal3	*χ*^2^ = 3.96, *p* = 0.047*	*χ*^2^ = 3.57, *p* = 0.059	*χ*^2^ = 4.47, *p* = 0.034*	*χ*^2^ = 4.03, *p* = 0.045^a^

^a^*p* < 0.05.

In short, of the 16 tests carried out, five produced significant results. Most of these significant results and ‘near misses’ occur when the inferred alligatoroid dispersal events are compared to the expected values (table 3), suggesting that the signal for reduced transoceanic dispersal in this clade is stronger than that for a greater number than expected of transoceanic dispersals among 'non-alligatoroid crocodylians'.

## 4. Discussion

### 4.1. Neosuchian biogeographic history

There is considerable consensus in the results of our analyses that allow us to propose a new biogeographic history for neosuchians and to evaluate previous hypotheses for the clade, although we acknowledge that agreement is not complete. After their origins in western Laurasia around 200 Ma, neosuchians began to disperse to eastern Laurasia and Gondwana. Early branching non-pelagic neosuchians are relatively rare in the fossil record and are known mostly from the families Goniopholididae, Atoposauridae and Paralligatoridae. The potentially oldest taxon is the goniopholidid *Calsoyasuchus valliceps* from the Early Jurassic of Arizona, USA [76], although its position within Neosuchia has been questioned [77]. Pelagic early branching neosuchians are known almost exclusively from the Middle or Late Jurassic, making it difficult to pinpoint an exact geographical origin, with few studies available on the topic at present. Thus, although both dated trees suggest that Neosuchia originated approximately 200 Ma [38], it seems that much of the clade's early evolutionary history remains obscure owing to poor sampling. Nevertheless, our biogeographic results most frequently place the origin of Neosuchia in Europe (BGB) and eastern North America (BALE), around 200 Ma (table 2), suggesting that the group originated somewhere in northwestern Pangaea in the latest Triassic. At this time, North America and Europe were still in relative geographical proximity as part of the supercontinent Pangaea, with Europe and eastern North America sharing lacustrine and coastal habitats [49,78]. However, some caution is required because of the recent discovery of *Burkesuchus*, an early branching mesoeucrocodylian from the Late Jurassic of Chile that lies outside clades such as Goniopholididae, Atoposauridae and Paralligatoridae [22]. Another potential paralligatoroid, *Batrachomimus,* is known from the Jurassic of Brazil [79]. Incorporation of *Burkesuchus* and *Batrachomimus* into future phylogenetic and biogeographic analyses has the potential to modify both the dating of key early divergence events, and the estimation of Neosuchia's area of origin.

Similar to Neosuchia, the ancestral area for Goniopholididae is most frequently estimated as Europe (BGB) and middle and eastern North America (BALE) in our analyses. Our BGB results suggest the movement of species from Europe to the Americas during the Early Jurassic, with subsequent dispersal to southeast Asia either from Europe (G22) or North America (SB21) during the Middle or Early Jurassic, respectively. This is in contrast to previous studies that have postulated North America as the area of origin, with subsequent radiations to Europe and Asia during the Cretaceous [30]. Although our BALE results continue to support a North American origin, the proposal that the subsequent dispersals occurred in the Cretaceous is no longer tenable.

Our results provide no clear conclusion with regards to the area of origin of Tethysuchia, as eastern North America (SB21, BGB), westernmost Asia (SB21, cal3, BALE) and eastern Europe (SB21, FBD, BALE) are all possibilities (table 2). One potential problem is that the earliest known tethysuchian, *Meridiosaurus vallisparadisi* [80], from the Late Jurassic of Uruguay, was not included in the Stockdale & Benton [39] supertree. The addition of *Meridiosaurus* to future analyses clearly has the potential to modify the estimated biogeographic origins of Tethysuchia. Despite these issues, there is some consensus, such as agreement on a probable dispersal event to North Africa during the Middle Jurassic (G22) or Early Cretaceous (SB21), which served as a centre for subsequent dispersals to southern Africa, Europe, South America and North America throughout the Cretaceous, facilitated by the marine adaptations and high saltwater tolerance of many tethysuchian taxa [81,82]. Within Tethysuchia, it has been suggested that vicariance played a strong role (in particular with regards to the break-up of Gondwana) in the evolution of *Sarcosuchus* and Pholidosauridae [21], but this cannot be verified with our current datasets because of sampling issues.

Most of our analyses infer the ancestral area of Dyrosauridae as North Africa (all BALE results, and SB21 BGB), with a minority supporting eastern North America (G22, BGB) (table 2). A North African origin has also been suggested by the majority of previous studies [17,83,84] and is consistent with the recent discovery of the oldest member of the group, *Brachiosuchus kababishensis* from the Campanian of north-central Sudan [84]. Given that the latter species is not included in either of the phylogenetic datasets we used, it provides some independent support for our conclusions. Thus, a North African origin of Dyrosauridae during the early Late Cretaceous seems likely, with several subsequent transoceanic dispersals to and from both North and South America facilitated by the apparently high degree of saltwater tolerance in this group [81,82].

Previous work suggested that Eusuchia originated in Gondwana, if Paralligatoridae and therefore *Batrachomimus* are considered eusuchians [24]. However, the majority of our results indicate that this clade originated in Europe or the surrounding waterbodies during the latest Jurassic/Early Cretaceous. Exceptions to this indicate eastern North America and the Arabian Peninsula as alternative areas of origin (table 2). Thus, conservatively, the origin of Eusuchia can be traced to the European archipelago and/or the surrounding coasts of the Tethys Ocean. The oldest-known eusuchians are European (depending on the phylogeny, either *Portugalosuchus azenhae* from the late Cenomanian of Portugal [23], or *Hylaeochampsa vectiana* and *Turcosuchus okani* from the Barremian of the UK and Turkey, respectively [26]), supporting a European origin, as do putative hylaeochampsid remains from the Middle Jurassic of the UK [85]. Given our estimated area of origin, and several Early Cretaceous remains from Asia [25], it is likely that there was a substantial eastward dispersal of eusuchians during the Early Cretaceous [26].

Earlier studies proposed that Crocodylia originated in the circum-Tethyan region/Laurasia [23,40,86] during the Early or early Late Cretaceous, followed by dispersal to North America shortly thereafter. Strong links persisted during the Late Cretaceous and Palaeocene between North American and European, and European and African, crocodylian faunas [7,13,23]. This scenario is supported by our analyses, with Europe and North America as the estimated areas of origin for Crocodylia.

Numerous earlier works suggested that Alligatoroidea [87], Alligatoridae [2,18,36,88], and Alligatorinae [36,89,90], originated in North America, although Europe has also been proposed for Alligatoroidea and Globidonta [91]. The results of our analyses consistently support the majority view, estimating an origin in middle North America (or close to it) for Alligatoroidea, Globidonta, Alligatoridae and Alligatorinae (table 2). All our results require at least one dispersal event from North America to Europe (and back) during the Late Cretaceous, and a second one from North America to central/southeast Asia during the Late Cretaceous. These estimates are in line with most of the established literature, which postulates at least two dispersal events from North America to Asia, one during the Late Cretaceous and one during the Palaeocene/Neogene [87], and repeated dispersals of alligatorids between North America and Europe [6]. Such intercontinental dispersals among alligatoroids might be regarded as unexpected given their lower saltwater tolerance [2]: however, alligators seemingly adapt better to cold, dry climates than crocodylids [88,92], potentially facilitating dispersal across higher latitude land routes, such as the Thulean land bridge, in particular during the Palaeocene–Eocene Thermal Maximum [7].

The biogeographic history of Caimaninae has received detailed attention and is relatively well documented [18,93–96]. Although most extant caimans are known from Central and South America, their area of origin was most probably North America: the latter has yielded remains of the earliest-known caimanine taxon, *Bottosaurus* (and potentially *Brachychampsa*), which was present before contact between the two continents was established [94]. After the emergence of Caimaninae during the middle Late Cretaceous, there were probably multiple independent dispersal events between North and South America around the K-Pg boundary [18,93,95,96], as well as multiple transoceanic dispersal events between east Asia and South America (potentially via North America) during the Cretaceous (G22) or early Palaeogene (SB21). Such dispersals were potentially facilitated by island chains between North and South America when these areas were not in direct contact [16]. The North American caiman range began to contract during the Eocene in response to lower temperatures, eventually resulting in regional extinction [97,98]. Our results support this scenario, with most estimating an origin in middle North America, followed by dispersal to northern South America (including Central America) where the group radiated (G22 BGB and all SB21 analyses).

All our analyses agree on reconstructing Europe as the ancestral area of Diplocynodontinae, which is in partial disagreement with most previous studies, as detailed below. The oldest-known species is *Diplocynodon remensis* from the Thanetian of France [99], and this study postulated North America and Europe as the most likely areas of origin for diplocynodontids. The largest problem with the European origin hypothesis is the current lack of other early branching Early Cenozoic alligatoroids from which diplocynodontids could have evolved [100]. This has led some authors to propose Asia as the area of origin instead [101]. This ‘gap’ is also apparent in our data, as both trees show a long ghost range leading to Diplocynodontinae, no matter which time-scaling method is employed. The area of origin of this ghost lineage is either specifically North America (SB21) or less precisely Laurasia (G22). Nonetheless, a European origin for Diplocynodontinae, with its early evolution obscured by poor sampling of the fossil record, is the most defensible biogeographic hypothesis at present.

According to our analyses, Crocodyloidea probably originated in middle North America, which supports previous studies that postulated a North American or Asian origin for the clade, with a subsequent dispersal to Europe [102]. Although *Jiangxisuchus nankangensis* from the Late Cretaceous of China [103] is currently the oldest-known crocodyloid specimen, the earliest-branching crocodyloid species in both trees is *Prodiplocynodon langi* from the Maastrichtian of North America [2,104]. Accordingly, both trees suggest a subsequent dispersal (probably transoceanic, given that potential land bridges are too far north: see [42, S3]) to Asia during the Late Cretaceous, and from there to Europe (SB21) or North Africa (G22).

Our results are discordant with regard to Crocodylinae, although all estimated areas of origin are located on the African (G22 distance-based BGB, all SB21 analyses) or Asian (G22 BALE, and unconstrained BGB analyses) landmasses (table 2). The oldest specimen is difficult to determine, but is potentially *Crocodylus megarhinus* from the Rupelian of Egypt (as resolved in the G22 phylogeny), although it has also been identified as a non-crocodyline crocodyloid [105,106]. Most recent studies, and fossil discoveries, have tended to support Asia/Australasia as the ancestral area for Crocodylinae [1,19], with subsequent transoceanic dispersal to North America and then South America during the Miocene/Pliocene [2,95,107]. These results are echoed by the SB21 analyses. However, several studies have also suggested a radiation of *Crocodylus* elsewhere, such as from Africa to the Americas prior to the re-invasion of Africa [33,34] which our results cannot corroborate at present. Mekosuchinae (only present in the SB21 phylogeny) is an exclusively Australian clade and most likely originated there (table 2), although an Asian origin has been suggested recently [108].

The biogeographic origins of Tomistominae are ambiguous. This group is estimated to have appeared first either in Europe (BALE) or east/southeast Asia (BGB) based on the G22 phylogeny. By contrast, previous work has suggested the Mediterranean/east Atlantic/western Tethys region as the potential area of origin, followed by a number of dispersals to North America, Africa and Asia [20,32,109], including several transoceanic dispersals between east Asia, Europe and North Africa during the Eocene. This western Tethys/eastern Atlantic hypothesis receives some direct support from the fossil record: two of this clade's oldest potential members are *Maroccosuchus zennaroi* from the Ypresian of Morocco [32] and *Kentisuchus spenceri* from the Ypresian of the UK and France [109]. However, *Maroccosuchus* is placed as an early branching crocodyloid in the G22 trees [38]. Clearly, more work on the phylogenetic relationships of early-branching tomistomines (and the integration of the results into supertrees such as SB21) is required in order to make progress with this issue. Nevertheless, we note that our proposed European origin of this clade is not necessarily incompatible with the western Tethys/eastern Atlantic hypothesis.

Our analyses estimate the ancestral area of Gavialoidea as Europe (all SB21 trees), eastern North America (G22, BALE) or an area between the latter regions (G22, most BGB analyses) during the Late Cretaceous, when Europe and eastern North America were still in relatively close proximity [110]. Gavialinae cannot be defined in the SB21 tree, but our G22 results suggest that this clade probably originated in North Africa/Europe. Our analyses indicate several dispersals between Africa and North and South America, and also from Africa to India (the latter being previously proposed by Martin *et al.* [111]). All analyses suggest several transoceanic dispersal events between Europe, North Africa and eastern North America during the Late Cretaceous and Palaeogene. This supports suggestions that early gavialoids were probably saltwater tolerant, facilitating long distance transoceanic dispersals [2,35,36,95,112]. Recent phylogenetic analyses have begun to reconcile the differences between morphological and molecular studies and placed tomistomine and gavialoid taxa in different positions [108,113], which could impact dispersal hypotheses.

### 4.2. The relative roles of vicariance and dispersal in crocodile biogeography

#### 4.2.1. Vicariance versus dispersal?

Earlier quantitative studies proposed that vicariance, driven by continental fragmentation, played a strong role in eusuchian biogeography during the Cretaceous [7,15]. More recently, however, dispersal has been supported as the dominant explanation, at least in crocodyline biogeography [19]. These two results are not necessarily mutually exclusive, however: an earlier and phylogenetically ‘deeper’ vicariance pattern might have been imposed on Eusuchia as its early branching lineages radiated against the backdrop of Pangaean fragmentation during the Mesozoic [78,110], followed by a more recent and phylogenetically ‘shallower’ set of dispersals that are most clearly manifested in smaller clades, such as Crocodylinae. Our results also support important roles for both vicariance and dispersal, although the identification of specific examples of the former, and matching them to palaeogeographic history, has proved problematic. The DEC + J model incorporating both vicariance and founder-event speciation [10,75] was the best fit to our data in the BGB analyses. Nevertheless, inspection of the ancestral area estimations has not revealed definitive examples of putative vicariance events that match predictions derived from palaeogeography. Although this might appear anomalous, we interpret this as potentially reflecting the impact of noise in our dataset created by factors such as an incomplete fossil record, taxonomic sampling, phylogenetic errors and so on. In particular, it should be noted that a BGB-based set of ancestral area estimations might find best solutions that require ancestors to occupy two or more areas and their daughter species to occupy single areas or subsets of those ancestral areas—such a scenario would result in models that incorporate vicariance (i.e. DEC and DIVALIKE) being favoured over those that exclude this process (i.e. BAYAREALIKE). However, noise or missing data might still result in a failure to match these putative vicariance events to palaeogeographic history. For example, suppose palaeogeographic history includes a supercontinent ABCD that fragments into two areas, A and BCD, followed by BCD splitting into areas B and CD, and finally C and D separate. Organisms with ancestral populations that lived in ABCD could potentially acquire a phylogeny with a (A(B(C,D))) vicariance pattern. In addition, suppose that one of the following also happens: the fossil record has not preserved the daughter lineages living in areas B and C; these lineages have not been sampled in a phylogenetic tree; and/or these lineages have been sampled in the tree but placed in the wrong relationships. Any of these problems could cause BGB to estimate that the ancestral node in question only occupied area A + D, followed by cladogenesis into two daughter lineages living in areas A and D, respectively. Thus, we have an AD ancestral area estimation leading to an inference of A/D vicariance, when in fact the correct result would be an ancestral area of ABCD followed by A/BCD vicariance. If we then attempt to identify an A/D vicariance event in palaeogeographic history, we might find that no such event is known, or even that such an event has occurred but does not match the timing predicted from our dated phylogeny. In short, the result that DEC + J is the best fitting model supports an important role for vicariance in neosuchian biogeographic history, but it appears that better fossil occurrence data and/or more comprehensive phylogenetic sampling is needed in order to match particular area fragmentation events to phylogenetic events.

Whatever the relative importance of vicariance, it is clear from our results that a considerable proportion of neosuchian biogeographic history has been dominated by dispersal, resulting in a number of founder-event speciation events. The nature of these dispersals, particularly with regard to overland routes versus transoceanic crossings, is discussed in more detail below.

#### 4.2.2. Transoceanic dispersal and saltwater intolerance

**Figure 2.**
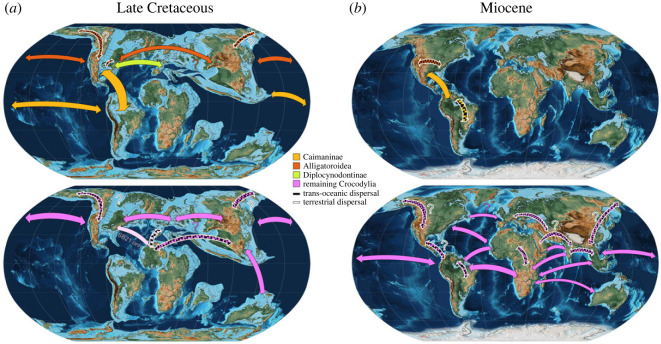
Potential terrestrial and transoceanic dispersal routes during neosuchan history. Maps show putative dispersal routes for Alligatoroidea (top) and remaining Crocodylia (bottom), during the Late Cretaceous (*a*) and Miocene (*b*). Chequered arrows indicate terrestrial dispersal routes. Question marks indicate putative terrestrial dispersal routes as alternatives to transoceanic ones. Arrow width indicates frequency of dispersal. ‘SB21 only’ signifies that the trans-oceanic dispersal was only suggested by the results of the Stockdale & Benton [39] tree. The maps are based on a reconstruction of the Earth 80 Ma and 16 Ma, respectively created in GPlates 2.0.0 [50,51] using the PALEOMAP PaleoAtlas [49].

One issue that complicates the reconstruction of neosuchian biogeography is the substantial difference in saltwater tolerance between clades, combined with the absence of direct fossil evidence for the soft tissue structures (situated mainly on the tongue) associated with such tolerance [77,114]. It has been suggested that saltwater tolerance arose multiple times throughout crocodylomorph history [36,77]. Our results indicate relatively high rates of dispersal (especially transoceanic dispersal) in most neosuchian groups, with a slightly higher frequency among Crocodylinae (consistent with the results of Nicolaï & Matzke [19]; figure 2)*.* Alligatoroidea is the only extant group without salt-excreting glands [77,114], and it is therefore predicted that this group should show a substantially reduced frequency of putative transoceanic dispersal events. As predicted, BGB estimates exhibit significantly fewer events among alligatoroids than expected from a non-skewed distribution (see above). These results underscore the relative saltwater intolerance of alligatoroids throughout their evolutionary history. Moreover, the few transoceanic events that are inferred for alligatoroids display a potentially meaningful phylogenetic clustering and/or are subject to alternative interpretations. Most putative transoceanic dispersal events within Alligatoroidea occur in Caimaninae in the G22 analyses, with only one or two such events among early alligatoroids. Similarly, the SB21 analyses propose two transoceanic dispersals within Caimaninae, one within Alligatorinae, and one in Diplocynodontinae. Although tentative at this stage, this distribution might indicate some retention of ancestral saltwater tolerance in the earliest alligatoroids, followed by an early loss of tolerance in most lineages, and reacquisition of tolerance in one or more caimans. The transoceanic dispersals among caimans, between North and South America, might also have been facilitated by intervening islands that enabled a series of shorter marine crossings rather than a single long one [16,115] (see [42, S3]). Furthermore, as noted in §4.1, it is possible that Late Cretaceous/Palaeocene dispersals of alligatoroids from North America to Asia and Europe occurred via higher latitude landbridges rather than lower latitude transoceanic crossings: this could have been more feasible for alligatoroids than other crocodile groups, given the former's higher tolerance for cooler conditions [116,117]. Thus, some of our inferred transoceanic dispersals might have occurred by longer land routes: if correct, this would reduce the number of transoceanic dispersals completed by alligatoroids even further.

Finally, as noted above, the results of our biogeographical estimations are more uniform for Alligatoroidea than for other, saltwater tolerant, groups (table 2). It is conceivable, therefore, that the lower frequency of transoceanic dispersal within Alligatoroidea has placed more constraints and uniformity on its distributions, leading to less ‘noise’ with regards to ancestral area estimation. By contrast, the higher frequency of transoceanic dispersals among non-alligatoroid crocodylian groups, such as Crocodylinae, has potentially allowed very complex networks of biogeographic relationships to evolve, and these are presumably more difficult to disentangle with currently available analytical techniques and phylogenetic/fossil record data.

## 5. Conclusion

In this study, we present the most comprehensive analysis of neosuchian historical biogeography attempted to date. We used a multivariate *k*-means clustering approach to establish 10 distinct geographical areas as a basis for formalizing neosuchian distributions. For our biogeographic analyses, we used two contrasting phylogenetic frameworks, each of which had been time-calibrated using the cal3 and FBD approaches [38,39]. We employed two different biogeographic methods, BGB [9,10] and BALE [12] to analyse our combined phylogenetic and distributional datasets.

The centre of origin for Neosuchia is estimated as lying somewhere within northwestern Pangaea, where the oldest putative species have also been found, with subsequent dispersals to the rest of Pangaea. Tethysuchian origins remain obscure, but we estimate a North African ancestry for Dyrosauridae, in agreement with most previous work. Eusuchia is estimated to have a European origin, in contrast to several prior studies, although our conclusion does align with the current fossil evidence. Similar to previous analyses, the area where Crocodylia first arose is estimated as either Europe or North America. Our results estimate origins for Alligatoroidea and Crocodyloidea in North America, whereas the origin of Gavialoidea is less conclusive (although this probably included eastern North America, Greenland and/or Europe). These results are generally consistent with other proposals for the latter two clades in the literature [87,102], including their subsequent dispersals to Asia, Europe and Africa.

Vicariance seems to have played a less important role in the evolutionary history of Neosuchia than first thought [15,21]. It was potentially more important during the earlier phases of the clade's evolution as Pangaea fragmented during the Jurassic and Early Cretaceous, whereas dispersal became more important from the Late Cretaceous onwards. Reconstructing neosuchian biogeographic patterns is complicated further by variation in saltwater tolerance among its various subclades—Alligatoroidea is saltwater intolerant, whereas the other neosuchian clades show varying degrees of saltwater tolerance, including fully marine forms in early occurring families such as Dyrosauridae. These different physiologies appear to have left their mark on the historical biogeography of alligatoroids and ‘other neosuchians’, with the former displaying significantly fewer instances of inferred transoceanic dispersal.

It is clear that current datasets remain inadequate to accurately constrain several key aspects of neosuchian biogeographic evolution. Many extinct taxa remain to be integrated into existing phylogenies, and major topological differences persist between the phylogenies generated by different datasets and methods. Moreover, there are multiple different approaches to the estimation of historical biogeographic patterns, and uncertainties remain in how they perform relative to each other, their accuracy under different conditions, and their abilities to cope with missing data and uneven sampling. Nonetheless, the observation that our BGB analyses have been able to detect the impact of saltwater tolerance/intolerance is encouraging and suggests that our current methods and data are capable of capturing at least some genuine aspects of neosuchian biogeographic history.

## Data Availability

Data is available from the Dryad Digital Repository: https://doi.org/10.5061/dryad.59zw3r2d2 [42].
